# Constructing Core-Shell Co@N-Rich Carbon Additives Toward Enhanced Hydrogen Storage Performance of Magnesium Hydride

**DOI:** 10.3389/fchem.2020.00223

**Published:** 2020-04-07

**Authors:** Ke Wang, Qibo Deng

**Affiliations:** ^1^School of Materials Science and Engineering, University of Shanghai for Science and Technology, Shanghai, China; ^2^Institute for New Energy Materials & Low-Carbon Technologies, School of Materials Science and Engineering, Tianjin University of Technology, Tianjin, China; ^3^Research Institute for Structure Technology of Advanced Equipment, School of Mechanical Engineering, Hebei University of Technology, Tianjin, China; ^4^Key Laboratory of Advanced Energy Materials Chemistry (Ministry of Education), College of Chemistry, Nankai University, Tianjin, China

**Keywords:** magnesium hydride, hydrogen storage performance, core-shell (C-S)nanostructures, carbon additives, dehydrogenation kinetics

## Abstract

Magnesium hydride (MgH_2_) is regarded as a promising solid-state hydrogen storage material, on account of its moderate price and high gravimetric capacity. However, MgH_2_'s inferior kinetic of hydrogen release impedes its widespread application. In this work, we use core-shell Co@N-rich carbon (CoNC) additive as catalysts to ameliorate the performances of MgH_2_. The surface morphologic structures and hydrogen desorption kinetics of different MgH_2_-CoNC composites are systematically studied. We find that MgH_2_-5 wt% CoNC with carbon contents of 17% (CoNC0) composites exhibit better hydrogen desorption performance. At 325°C, the MgH_2_-5 wt% CoNC0 composites can release up to 6.58 wt% of H_2_ in 5 min, which is much higher than 0.3 wt% for pure MgH_2_. Our results demonstrate that importing the core-shell structured catalysts can effectively enhance the hydrogen release kinetics.

## Introduction

Low-cost manufacturing, safe storage, and transportation, as well as the effective conversion of hydrogen are the basic requirements for the realization of the large-scale application of hydrogen energy (Baykara, 2018; Abe et al., 2019; Li A. et al., 2019; Staffell et al., 2019; Wei et al., 2019; Xu et al., 2019). Therefore, it is essential to explore a novel and high-capacity hydrogen storage material. Magnesium hydride (MgH_2_) has been considered as a promising hydrogen storage material due to its high hydrogen storage amount of 7.6 wt% and high volumetric hydrogen storage density of 110 kg m^−3^ (Aguey-Zinsou and Ares-Fernandez, 2010; Jeon et al., 2011; Shao et al., 2018; Yartys et al., 2019). However, the high hydrogen desorption temperature, sluggish kinetics, and thermodynamics performances of MgH_2_ have impeded its further applications.

To enhance the hydrogen adsorption kinetics properties of Mg/MgH_2_ materials, three different strategies have been investigated to decrease the dehydrogenation temperature: nano-crystallization (Zhu et al., 2011; Lin et al., 2016; Li et al., 2018), alloying with transition metal (Rusman and Dahari, 2016; Wang et al., 2016; Zhong and Xu, 2019), and catalyst additives (De et al., 2016; Zhang et al., 2018; Liu et al., 2019b; Wang et al., 2019; Wang K. et al., 2019; Zhou et al., 2019a,b). Several papers have demonstrated that using proper catalysts is more convenient for practical applications (Zhang et al., 2017, 2019; Huang et al., 2018; Valentoni et al., 2018; Chen et al., 2019; Li B. et al., 2019; Hu et al., 2020). Among the catalyst additives, the prominent catalytic influences of Co metal on enhancing the hydrogen desorption properties of MgH_2_ have been reported in previous literature (Mao et al., 2010; Novakovic et al., 2010; Verón et al., 2013; Liu et al., 2018, 2019a). Novakovic et al. have reported that the higher number of d-electrons in Co metal has made it superior to Ti in destabilizing MgH_2_ (Novakovic et al., 2010). Mao et al. proved that the dehydrogenation temperature was lower and the adsorption/desorption kinetics could be enhanced by adding CoCl_2_ catalyst (Mao et al., 2010). MgH_2_-Co mixture was reported showing better hydrogen storage properties and high-rate hydrogen adsorption/desorption (Verón et al., 2013). Liu et al. have demonstrated that Co@CNTs nano-catalyst doped into MgH_2_ played an essential role in improving its hydrogen storage properties (Liu et al., 2018). A novel bi-metallic Co/Pd@B-CNTs catalyst was also reported recently showing excellent catalytic effects of MgH_2_ at low temperatures (Liu et al., 2019a). Based on literature, the mixture of Co metal and carbon material exhibits effectively catalytic function. It is well-known that the morphology and micro-structure of materials is one of the significant factors to further improve its physical or chemical performance. In comparison with the bulk structure, the core-shell structure exhibits much higher specific surface area for exposed active sites and the more electronic interaction of core and shell material. Different constituent of core and shell can be modulated as a parameter to exhibit the optimal synergistic effect. Herein, we introduced the core-shell Co@N-rich carbon hybrids as catalyst additives into MgH_2_ system to effectively improve the hydrogen desorption performances of MgH_2_. The carbon shell can protect Co core from oxidation and aggregation. The core-shell structure could further significantly enhance the intimate interface between Co@C and MgH_2_, providing more active “catalytic sites” and hydrogen “diffusion channels” to reduce the dehydrogenation temperature. Such benefits of additives with core-shell structure then improve the dehydrogenation kinetics of MgH_2_. Our study also compared the effect of different carbon contents and found that the MgH_2_-5 wt% CoNC with the carbon contents of 17% (CoNC0) composites had the lowest dehydrogenation temperature and best dehydrogenation kinetic properties.

## Materials and Methods

The chemical agents used in this work were purchased from Adamas. Core-shell Co@N-rich carbon hybrids were synthesized according to our previous work (An et al., 2017). The obtained sample with the carbon contents of 17% was designated as CoNC0, and the sample with the carbon amount of 25% was designated as CoNC1. The purchased MgH_2_ was mixed with 3 or 5 wt% of CoNC0 and CoNC1 hybrids through ball-milling at room temperature for 5 h at 450 rpm under 2 MPa H_2_ pressure. The mass ratio of big or small balls and powder was about 40:1.

The surface morphological structures of CoNC and various MgH_2_-CoNC composites were determined by transmission electron microscopy (TEM). The thermal decomposition of various MgH_2_-CoNC composites was studied on differential scanning calorimetry (DSC) and temperature programmed desorption (TPD). The test conditions of DSC measurement were as follows: heating rate of 2, 5, 10, and 15°C min^−1^, shielding and sweeping gas of high-purity Ar with 30 ml min^−1^ flow rate, respectively. As for TPD, the Ar flow rate was 35.1 ml min^−1^ and the measured temperature was 50–500°C. The isothermal hydrogen desorption properties were characterized by a self-made Sieverts-type instrument under an initial pressure of 0.05 MPa hydrogen at 275, 300, and 325°C, respectively. After complete dehydrogenation, the pressure increased to 0.08 MPa.

## Results and Discussion

**Figure 1:**
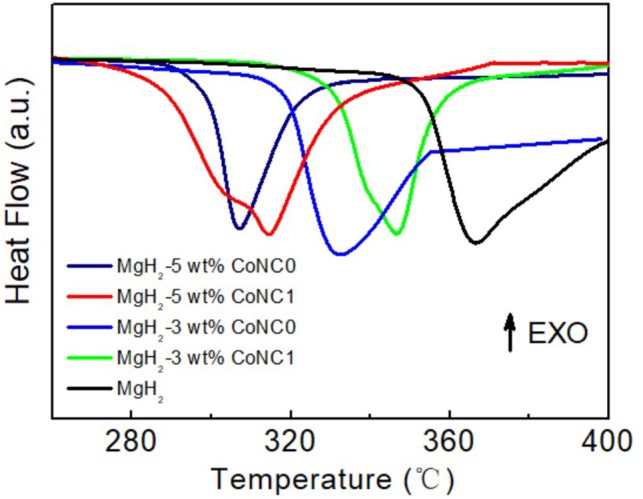
DSC curves of the pure MgH_2_, MgH_2_-3 wt% CoNC0, MgH_2_-5 wt% CoNC0, MgH_2_-3 wt% CoNC1, and MgH_2_-5 wt% CoNC1 composites at a heating rate of 5°C min^−1^.

Figure 1 displays the DSC curves of pure MgH_2_ and various MgH_2_-CoNC composites to investigate the thermal decomposition properties at a heating rate of 5°C min^−1^. Obviously, the onset and hydrogen desorption temperatures of MgH_2_-CoNC composites are lower than that of pure MgH_2_, demonstrating that the addition of CoNC hybrids can improve the hydrogen desorption kinetics of MgH_2_. The value of the onset and hydrogen desorption temperatures for these MgH_2_-CoNC composites are listed in Table 1. The onset temperature suggests the dehydrogenation starting. As displayed in Figure 1, there is a broad hydrogen desorption peak during the heating process in MgH_2_, MgH_2_-3 wt% CoNC1, MgH_2_-3 wt% CoNC0, and MgH_2_-5 wt% CoNC1 composites, suggesting the sluggish hydrogen desorption kinetics. As for the remaining MgH_2_-5 wt% CoNC0 composites, there is only one sharp peak, located at 307°C, further implying the enhanced hydrogen desorption kinetics. Moreover, there are two endothermic peaks observed in the case of MgH_2_-3 wt% CoNC1 and MgH_2_-5 wt% CoNC1 samples. The identification of two peaks may be due to bimodal particle size distribution formed during ball-milling. This issue can be reduced when increasing the ball-milling time.

**Table 1:** The onset and peak temperatures of the MgH_2_ and various MgH_2_-CoNC composites.

**Sample**	**Onset temperature (°C)**	**Peak temperature (°C)**
MgH_2_	344	367
MgH_2_-3 wt% CoNC0	313	332
MgH_2_-5 wt% CoNC0	285	307
MgH_2_-3 wt% CoNC1	312	346
MgH_2_-5 wt% CoNC1	269	314

**Figure 2:**
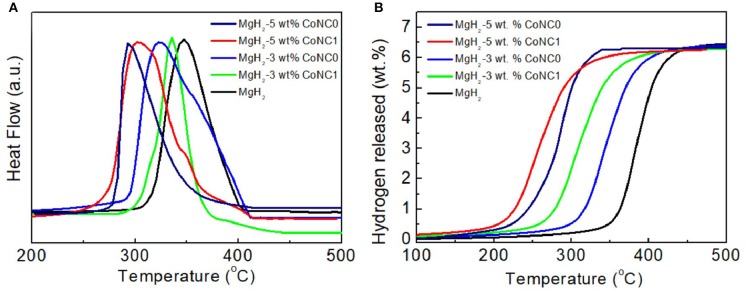
The TPD plots **(A)** and the corresponding H_2_ desorption amount curves **(B)** of the pure MgH_2_ and MgH_2_-CoNC composites.

In order to further illustrate the impacts of the CoNC additives on the hydrogen desorption properties of MgH_2_, TPD measurements have been conducted (Figure 2). There are two hydrogen desorption peaks of the MgH_2_-5 wt% CoNC1 composites in the pyrolysis procedure, which can be ascribed to the uneven distribution of the particles after the addition of CoNC1 hybrids (Figure 2A). The peak temperatures of MgH_2_-5 wt% CoNC0, MgH_2_-5 wt% CoNC1, MgH_2_-3 wt% CoNC0, MgH_2_-3 wt% CoNC1, and pure MgH_2_ are 293, 304, 324, 336, and 348°C, respectively. Obviously, the peak temperatures of the MgH_2_-CoNC composites are lower than that of pure MgH_2_. Similarly, the onset temperatures for the above four composites are lower than that of pure MgH_2_, illustrating the improved dehydrogenation kinetics. This observation from TPD results is consistent with the DSC results. Compared with the pure MgH_2_, the dehydrogenation amount of the four MgH_2_-CoNC composites was almost the same (Figure 2B), due to the fact that the CoNC hybrids are non-active materials for hydrogen adsorption. Among all the samples, the MgH_2_ with 5 wt% CoNC0 additives exhibits the decreased hydrogen desorption temperature. Therefore, after comprehensive analysis, MgH_2_-5 wt% CoNC0 composites have been regarded as the optimal material for hydrogen storage in this study.

**Figure 3:**
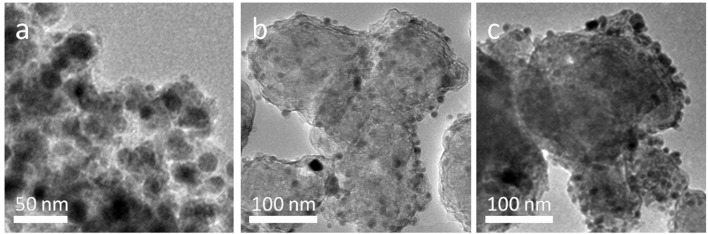
TEM images of CoNC0 **(a)**, MgH_2_-5 wt% CoNC0 composites after first dehydrogenation **(b)**, and MgH_2_-5 wt% CoNC0 composites after five dehydrogenation cycles **(c)**.

The surface morphological structures of CoNC0 additives and MgH_2_-5 wt% CoNC0 composites are characterized and the corresponding TEM images are shown in Figure 3. CoNC0 hybrids display a core-shell structure with a Co core (18 nm) coated with N-rich carbon shell. More details on core-shell structure of hybrids are available in our previous report (An et al., 2017). The TEM images of MgH_2_-5 wt% CoNC0 composites after first dehydrogenation and five cycles are also presented in Figure 3. The MgH_2_-5 wt% CoNC0 composites after first dehydrogenation (Figure 3b) showed irregular morphologies of accumulated nanoparticles (~30 nm in diameter). Likewise, after five cycles, the anomalous morphology and structure of the MgH_2_-5 wt% CoNC0 composites have been retained while the size of the nanoparticles have increased apparently (Figure 3c). The morphological changes after dehydrogenation can be explained by the disaggregation, spreading, nucleation, development, and re-separation procedures of the nanoparticles during hydrogen adsorption-desorption process. The interface migration, disintegration, and incorporation of various phases have been referred to this process, in which the formation of metal hydride would lead to the rapid increase of nanoparticle size after several hydrogen adsorption–desorption cycles.

**Figure 4:**
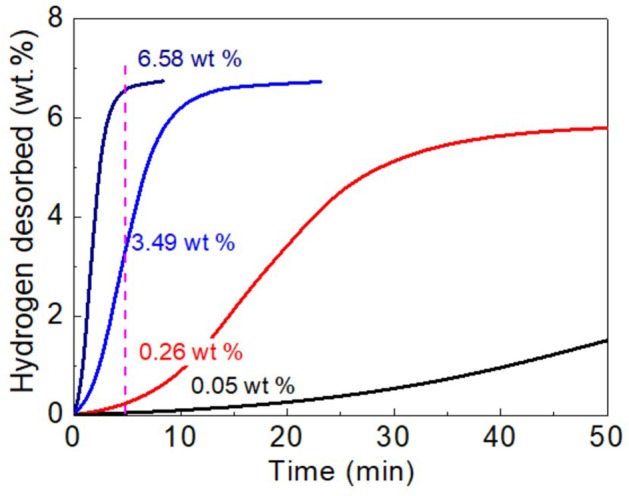
Hydrogen desorption kinetics curves of pure MgH_2_ at 300°C (black line), MgH_2_-5 wt% CoNC0 at 275°C (red line), MgH_2_-5 wt% CoNC0 at 300°C (blue line), and MgH_2_-5 wt% CoNC0 at 325°C (navy line).

To gain a deeper understanding on the enhanced dehydrogenation kinetics of MgH_2_-5 wt% CoNC0 composites, the hydrogen desorption kinetics plots at different temperatures (275, 300, and 325°C) are obtained (Figure 4). At the same temperature (300°C), the dehydrogenation amount of MgH_2_-5 wt% CoNC0 composites can reach up to 3.49 wt% in 5 min while the amount is only 0.05 wt% for pure MgH_2_. Even though the reaction time is extended to 50 min, the dehydrogenation amount of pure MgH_2_ reaches a value of 1.51 wt%, which is still lower than that of MgH_2_-5 wt% CoNC0 composites in 5 min. The slope for MgH_2_-5 wt% CoNC0 composites is much larger than that of pure MgH_2_, further demonstrating that the addition of CoNC0 hybrids has a prominent influence on the dehydrogenation kinetics of pure MgH_2_. As for MgH_2_-5 wt% CoNC0 composites, the dehydrogenation temperature has a significant impact on the hydrogen desorption amount. Specifically, the hydrogen desorption amount of MgH_2_-5 wt% CoNC0 composites at 325 and 275°C are 6.58 wt% and 0.26 wt% in 5 min, respectively, which increases nearly 25 times.

**Figure 5:**
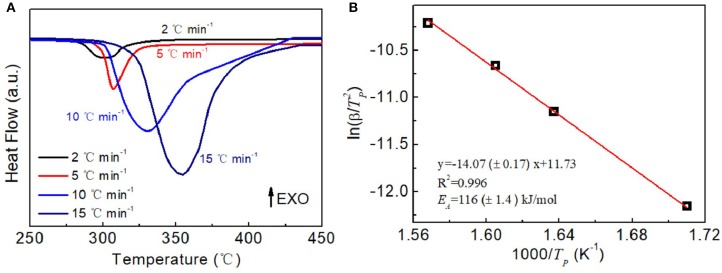
DSC **(A)** and the fitting Kissinger plots **(B)** of the MgH_2_-5 wt% CoNC0 at 2°C min^−1^, 5°C min^−1^, 10°C min^−1^, and 15°C min^−1^ heating rate.

The above results have further demonstrated that the CoNC0 additives could enhance the dehydrogenation kinetic performances of MgH_2_. Next, the activation energy of hydrogen desorption for MgH_2_-5 wt% CoNC0 composites is investigated by DSC measurements at various heating rate. There is only one endothermic peak of the MgH_2_-5 wt% CoNC0 composites at various heating rate (Figure 5A). The temperatures of hydrogen desorption process of MgH_2_-5 wt% CoNC0 composites are 298, 307, 330, and 354°C at a heating rate of 2, 5, 10, and 15°C min^−1^, respectively. The activation energy of MgH_2_-5 wt% CoNC0 composites is then calculated according to the following equation:

$$\begin{array}{l} -\frac{{\text{E}}_{\text{a}}}{\text{R}}=\frac{\text{d}\left[\text{ln}\left(\frac{\beta }{{{\text{T}}_{\text{P}}}^{2}}\right)\right]}{\text{d}\left(\frac{1}{{\text{T}}_{\text{P}}}\right)}. \end{array}$$

According to the fitting result, ln(β/${\text{T}}_{\text{P}}^{2}$) depends linearly on 1/T_P_, which is consistent with the Kissinger plot (Figure 5B). Based on the fitted slope of the Kissinger plot and the constant *R*, the activation energy *E*_a_ is determined to be 116 ± 1.4 kJ mol^−1^. The *E*_a_ value of MgH_2_-5 wt% CoNC0 composites is comparable to the values for the materials reported previously, such as MgH_2_-10 wt% CoB/CNTs (119 kJ mol^−1^) (Gao et al., 2017), MgNCG (137 kJ mol^−1^) (Liu et al., 2016), MgH_2_-TiN (144 kJ mol^−1^) (Wang et al., 2015), and MgH_2_-FeCl_3_ (130 kJ mol^−1^) (Ismail et al., 2014). These results further manifest the prominent effects of the Co@C additives on enhancing the dehydrogenation kinetics of pure MgH_2_. However, the *E*_a_ value of MgH_2_-5 wt% CoNC0 is higher than 53.4 kJ mol^−1^ of FeCo nanosheets, 67.64 kJ mol^−1^ of TiO_2_ nanosheets, 82.2 kJ mol^−1^ of ZrMn_2_ nanoparticle, and 99 kJ mol^−1^ of VNbO_5_ in the current literature (Valentoni et al., 2018; Yang et al., 2019; Zhang et al., 2019; Zhang M. et al., 2019). Lower activation energies could be due to the intrinsic catalytic activities of different additives with high surface energy. Comparing with many reported dopants, the simple preparation of CoNC composite and relatively inexpensive raw materials in our study may be advantageous to decrease the cost of product for practical application. The properties reported in this work could naturally be enhanced via optimizing the constituent structure, for example, the amount of the Co and the diameter of the core-shell nanoparticles, which will be the future research direction. It is also interesting to fabricate the core-shell structured catalyst additives with high catalytic activity in a simple preparation procedure for the hydrogen energy storage in a future study.

## Conclusion

To summarize, MgH_2_-x wt% Co@NC (Co@NC0 and Co@NC1) (*x* = 3, 5) composites were synthesized via ball-milling method. The microstructure, dehydrogenation kinetics of the MgH_2_-x wt% Co@NC composites and the influences of Co@NC additives on the hydrogen desorption kinetics of MgH_2_ materials are discussed. Based on the experimental results, the addition of Co@NC additives has promoted the hydrogen desorption kinetics of MgH_2_. In addition, the MgH_2_-5 wt% CoNC0 composite exhibits the lowest hydrogenation temperature and maintains a moderate dehydrogenation amount. The MgH_2_-5 wt% Co@NC0 composites generate 6.58 wt% hydrogen in 5 min at 325°C and 3.49 wt% hydrogen in 5 min at 300°C. Moreover, according to the Kissinger plot, the calculated *E*_a_ of the MgH_2_-5 wt% Co@NC0 composites is about 116 kJ mol^−1^, indicating that the Co@NC hybrids have effectively promoted the hydrogen adsorption kinetics of MgH_2_. Our work imports the core-shell microstructure to play a positive role on the hydrogen storage performance of magnesium hydride and then provides useful guidance for the future development of advanced materials for hydrogen storage.

## Data Availability Statement

All datasets generated for this study are included in the article/supplementary material.

## Author Contributions

KW performed the experiments, analyzed the data, and wrote the original draft. QD designed the experiments and reviewed manuscript. KW and QD revised manuscript. All authors discussed the results and commented on the manuscript.

### Conflict of Interest

The authors declare that the research was conducted in the absence of any commercial or financial relationships that could be construed as a potential conflict of interest.
